# Supplementary material for: Effects of Long-Term Heavy Metal Exposure on the Species Diversity, Functional Diversity, and Network Structure of Oral Mycobiome
Source: Microorganisms. 2025 Mar 7;13(3):622. doi: 10.3390/microorganisms13030622 (PMC11945009; doi:10.3390/microorganisms13030622)
Supplement: Supplementary file 1 [file microorganisms-13-00622-s001.zip › microorganisms-3449907-supplementary.pdf]

Supplementary Material

1 Supplementary Figures and Tables

1.1 Supplementary Figures

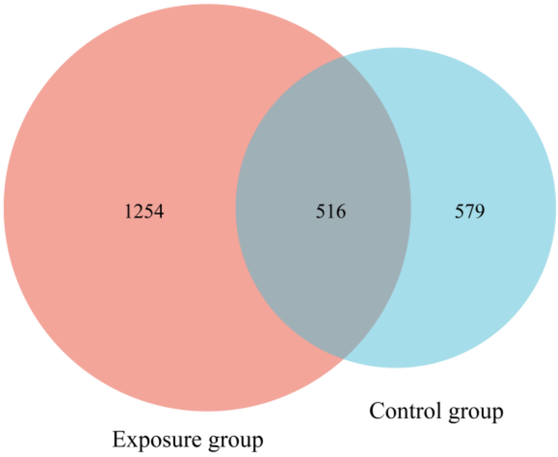

Supplementary Figure S1. Venn diagrams of oral fungal OTUs in two groups.

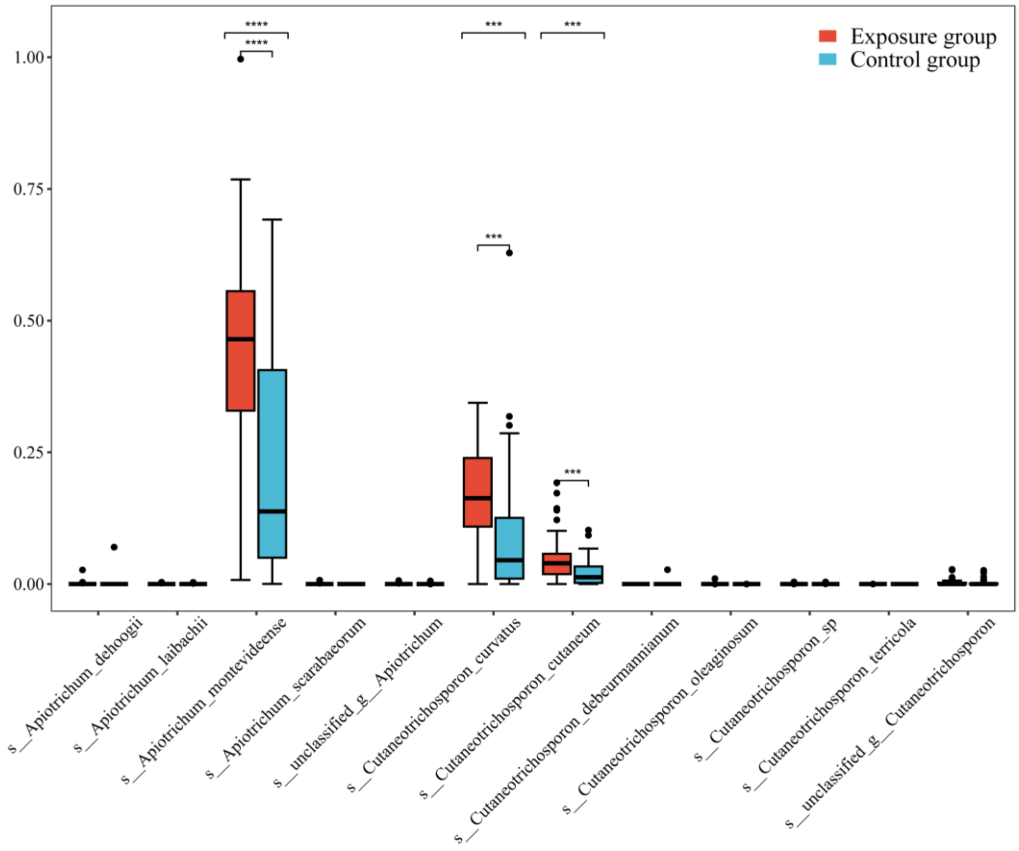

Supplementary Figure S2. Differential species of the genera *Apiotrichum* and Cutaneotrichosporon at the species level (***P*<0.0001, ****P* < 0.001, **0.001 < *P* < 0.01 and *0.01 < *P* < 0.05).**

1.2 Supplementary Tables

Table S1. Heavy metal content at sampling locations.

	Exposure group	Control group	*P*-value
Gender (M/F)	43/49	23/21	0.546
Age	59.99±6.47	59.36±6.32	0.365
Smoking	35 (38.0%)	19 (43.2%)	0.567
Alcohol	11 (12.0%)	7 (15.9%)	0.525

M is male, F is female.

Table S2. The trophic modes of species that differed in relative abundance at the genus level.

Phylum	Genus	Trophic mode	Guild
Basidiomycota	*Apiotrichum*	Saprotroph	Soil Saprotroph
Ascomycota	*Aspergillus*	Saprotroph	Undefined Saprotroph
Basidiomycota	*Cutaneotrichosporon*	Pathotroph	Animal Pathogen
Ascomycota	*Debaryomyces*	Saprotroph	Undefined Saprotroph
Ascomycota	*Wallemia*	Saprotroph	Undefined Saprotroph
Ascomycota	*Saccharomyces*	Saprotroph	Undefined Saprotroph
Basidiomycota	*Vishnia cozyma*	Pathotroph-Saprotroph-Symbiotroph	Fungal Parasite-Undefined Saprotroph
